# The effect of sleep restriction on cognitive performance in elite cognitive performers: a systematic review

**DOI:** 10.1093/sleep/zsab008

**Published:** 2021-01-13

**Authors:** Tim D Smithies, Adam J Toth, Ian C Dunican, John A Caldwell, Magdalena Kowal, Mark J Campbell

**Affiliations:** 1 Department of Physical Education & Sport Science, University of Limerick, Castletroy, Limerick, Ireland; 2 Lero, the SFI Centre for Software Research, University of Limerick, Castletroy, Limerick, Ireland; 3 Melius Consulting, Mount Hawthorn, Western Australia, Australia; 4 Centre for Sleep Science, School of Human Sciences, University of Western Australia, Crawley, Western Australia, Australia; 5 Coastal Performance Consulting, USA

**Keywords:** vigilance, cognitive flexibility, occupation, safety-critical, attention, sleep restriction

## Abstract

**Study Objectives:**

To synthesize original articles exploring the effects of sleep restriction on cognitive performance specifically for *Elite Cognitive Performers*, i.e. those who engage in cognitively demanding tasks with critical or safety-critical outcomes in their occupation or area of expertise.

**Methods:**

Backward snowballing techniques, gray literature searches, and traditional database searches (Embase, MEDLINE, Web of Science, Google Scholar, PSYCinfo, and SportDiscus) were used to obtain relevant articles. A quality assessment was performed, and the risk of training effects was considered. Results were narratively synthesized. Fourteen articles fit the criteria. Cognitive outcomes were divided into three categories defined by whether cognitive demands were “low-salience,” “high-salience stable,” or “high-salience flexible.”

**Results:**

Low-salience tests (i.e. psychomotor vigilance tasks & serial reaction tests), mainly requiring vigilance and rudimentary attentional capacities, were sensitive to sleep restriction, however, this did not necessarily translate to significant performance deficits on low-salience occupation-specific task performance. High-salience cognitive outcomes were typically unaffected unless when cognitive flexibility was required.

**Conclusions:**

Sleep restriction is of particular concern to occupations whereby individuals perform (1) simple, low-salience tasks or (2) high-salience tasks with demands on the flexible allocation of attention and working memory, with critical or safety-critical outcomes.

**Statement of Significance**

Sleep restriction is considered a significant concern to performance on cognitively demanding tasks within occupations that involve such tasks (i.e. pilots, air traffic controllers, surgeons, medical residents, emergency responders, process operators, athletes). However, no review to date has focused specifically on these populations, outlining the results of research exploring how the performance of these individuals is impacted by sleep restriction. Our review systematically searches for and narratively synthesizes the current literature to date within these populations, and outlines how cognitive tests and occupational tasks of different demands are differentially impacted by sleep restriction. Lastly, the review shows that more work is needed that examines the impact of sleep restriction on cognitive flexibility within these populations.

## Introduction

Optimal cognitive functioning is fundamental to performance within many work environments. In select safety-critical occupations, the ability to perform complex, cognitively demanding tasks within unpredictable circumstances is integral to operational success. Active military personnel [1], aviation pilots [2], air traffic controllers [3], emergency responders [4], surgeons and medical practitioners [5, 6], and process operators in potentially dangerous environments (i.e. mines, power plants, oil refineries) [7] are all examples of individuals involved in such safety-critical professions. Additionally, while elite athletes do not engage in safety-critical work, optimal cognitive functioning (i.e. attention, executive functioning, decision making) within time-constrained and unpredictable environments is often integral for elite performance [8, 9]. Individuals within these professions must exhibit cognitive expertise not normally present within the general population for operational success, given the complexities and cognitive demands embedded within the tasks involved. Individuals in some of the professions mentioned (i.e. athletes, pilots, air traffic controllers) have been shown to demonstrate enhanced cognitive performance compared to the general population not only within the context of their area of expertise, but also through laboratory testing [10–13], though see an article for Taylor and colleagues [14] for a contrary finding, particularly during task-switching, multitasking and attentionally demanding task paradigms. As a result of the aforementioned cognitive demands and the observed performance benefits these individuals may possess, we refer to them here collectively as *Elite Cognitive Performers* (ECPs).

Sleep quantity has been identified as a key moderator of cognitive performance [15–18]. To date, most sleep quantity research has concerned itself with total sleep deprivation (TSD; a total elimination of sleep obtained during a specified time period), primarily due to the time and cost efficiency of their designs [19]. However, TSD is uncommon ecologically, whereas sleep restriction (SR), referring to a moderate reduction in the amount of sleep across one or more nights (~2–6 h sleep obtained per night), is far more commonly experienced both by the general population [19] and by ECPs [20, 21]. The fact that SR is more frequently experienced than TSD, and that each affects human neurobiology differently [19], has led more recent work to specifically focus on understanding the effects of SR on cognitive performance. In addition to the reviews assessing the effects of SR on cognitive performance among youth [22] and adolescent [23] populations, experimental sleep dose–response studies, such as those conducted by Belenky et al. [24], Jewett et al. [25], Van Dongen et al. [26], and Banks et al. [27], have provided comprehensive insight into the effects of SR on cognition. The results of studies such as by Belenky et al. [24] and by Van Dongen et al. [26], as well as other experimental research, have informed the creation of biomathematical fatigue models, used in safety-critical environments to identify periods of risk and, guide mitigation, and maximize performance [28]. Recently, Lowe et al. [17], in a meta-analysis investigating the effects of SR on cognitive performance, found SR to impact “sustained attention” tasks more than increasingly complex tasks assessing performance in other cognitive domains across numerous populations and age groups. This finding corroborates those of Wickens et al. [29], who noted that simple cognitive task performance is more greatly impacted by sleep loss, as well as earlier seminal research outlining the comparatively greater effects of sleep loss on simple tasks [30].

That performance on simple tasks appears selectively hindered by SR initially seems counter-intuitive, as prefrontal cortex (PFC; integral to executive functioning) activation is decreased by sleep loss [31, 32]. However, imaging studies (using functional magnetic resonance imaging) have found strong evidence for increased recruitment of frontostriatal circuits and additional brain areas coinciding with the maintenance of performance during increasingly complex and engaging cognitive tests despite decreased PFC activation [31, 33–36]. Through this lens, simple attentional test performance tends not to receive similar compensation due to a lack of arousal, stemming from the low stimulus/salience nature of such tests [37, 38]. Recent work has suggested that these compensatory mechanisms function in a way so as to give preference to task information already present within working memory, helping to maintain focus and attentional strategy throughout the task (i.e. cognitive stability). However, the trade-off appears to be that the ability to alter this information within working memory (i.e. cognitive flexibility), necessary for when attention needs to be shifted when a task dynamic changes (as is common within real-world tasks), is impeded [38].

Despite the abovementioned literature outlining the effects of SR among general populations, it is less clear how SR affects the cognitive performance of ECPs or whether this group is differentially affected by SR. The importance of studying this group independently from the general population is three-fold. Firstly, optimal cognitive performance is arguably more important for ECPs than for the general population, as errors or inadequate performance can have critical outcomes, ranging from loss of competition for high-level athletes, to loss of life in safety-critical occupations. Numerous high-profile catastrophes have involved human errors linked to sleep loss, such as the fatal decision to launch the Space Shuttle Challenger in 1986. In the report on the *Presidential Commission on the Space Shuttle Challenger Accident* [39], it was stated that prior to an important teleconference regarding the decision to launch (a decision proving to result in seven casualties), “key managers obtained only minimal sleep the night before the teleconference” (p. G5), which may have led to poor judgement contributing to the fatal decision to launch. Another example is the pervasiveness of fatigue in aviation, where it is estimated that fatigue contributes to 4%–8% of aviation catastrophes [40].

Secondly, ECPs are at an increased risk of experiencing SR due to their occupational requirements. For example, sleep opportunity can be sparse and unpredictable throughout military combat operations, while other military-specific stressors, such as watch duty and field-based exercises, result in the frequent occurrence of SR [20]. Commercial pilots often have demanding schedules, are constantly exposed to rapid time-zone changes, and often must obtain night-time sleep in uncomfortable cockpit environments, resulting in regularly experienced SR. Rapidly changing work schedules are common for air traffic controllers, causing drastic reductions in sleep quantity, with some operating with as little as 2 h of sleep at times [41]. Irregular and demanding shift work schedules can lead to SR for emergency medical practitioners [42]. Finally, elite athletes can experience SR due to the timing and intensity of training and competition schedules, as well as air-travel requirements, especially when traveling over multiple time-zones [43].

Thirdly, contemporary literature has suggested that ECPs at a group level may demonstrate increased resistance to the effects of sleep loss on cognitive performance. For example, one study found a group of seven active-duty F117 fighter pilots to have greater baseline global cortical activation compared to nonpilots during a working memory task, which then positively correlated with performance on a flight simulator task after 37 h of TSD [44]; however, the authors advocated for further research on larger samples to validate such a finding. In reference to this, some authors have discussed the idea that naturally tolerant individuals to sleep loss may either “self-select” into, or that vulnerable individuals may “self-select” out of, active military professions due to the necessity of maintaining performance following sleep loss [21, 45, 46]. Similar theories have been posited to explain a lack of performance degradation following sleep loss among medical residents [47, 48]. It is noted that individual differences in tolerance to sleep loss within elite groups such as the U.S. Air Force are still present [49].

Together, the importance of optimal cognitive performance for ECPs, their increased risk toward experiencing SR, and their potential increased tolerance to the performance effects of SR at a group level, all make the study of the effects of SR on cognitive performance in ECPs worthwhile. To date, no attempt has been made to review the existing literature examining the effects of SR on the cognitive performance of ECPs. As a result, the purpose of this review is to synthesize and summarize the existing literature explicitly examining the effect of SR on cognitive and occupation-specific performance among ECPs.

## Methods

### Database search strategy

This review was not registered prior to its undertaking. Included articles did not have to be published in peer-review scientific journals to be considered. Articles included for the current review were obtained through an exhaustive systematic search, in accordance with the updated PRISMA guidelines [50]. Embase, MEDLINE (Ovid MEDLINE(R) and ePub ahead of print, in-process & other nonindexed citations, daily and versions(R)), Web of Science (Core Collection), and Google Scholar databases were searched, as the combination of these four databases presents superior sensitivity/specificity trade-off for systematic searches [51]. Subject-specific databases APA, PSYCinfo, and SportDiscus (both EBSCO host) were also queried to add further sensitivity to the search. Searches using these databases took place on January 27, 2020, except for Google Scholar, which took place the next day. The exact syntax used for each primary database can be found as supplementary file 1. The search strategy for each database involved identifying “key-words” (22 total) within titles and abstracts pertaining to the motor or cognitive abilities, or performance, and combining them with words pertaining to SR (five total), with the exclusion of words related to animal studies, clinical conditions, or reviews. Controlled vocabulary terms (MeSH/EMTREE) were explored and used as exploded terms (searching for the particular word as well as the more specific words that stem from it within the given organization system) where relevant in databases that allowed for them. Inbuilt database filters were used where available to remove studies specifically investigating nonhuman subjects, children, or the elderly; no date or language restrictions were enforced. TS performed the search and screening described.

**Figure 1.**
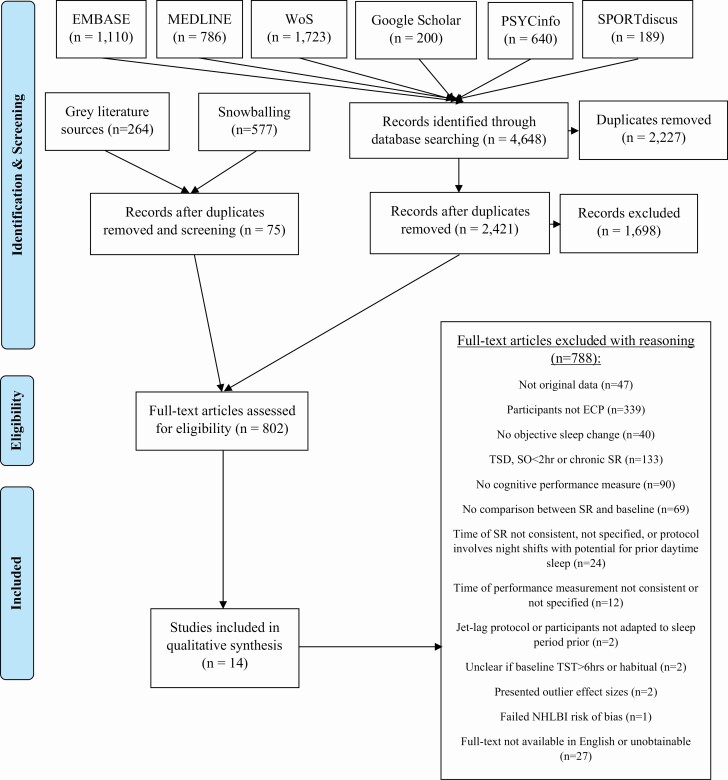
PRISMA flowchart outlining the eligibility and inclusion process for the current review.

All identified article references were extracted and exported into Endnote version 9.2 (Clarivate Analytics), except for those found via Google Scholar, where only the first 200 references (when searched by relevance; as per Bramer, Rethlefsen, Kleijnen, Franco [51]) were extracted. Overall, 4,648 articles were identified through this search process, with 2,421 remaining once duplicates had been removed (see Figure 1).

### Gray literature and backward snowballing

As some research concerning the effects of SR on performance among ECPs may not have been detected by the above database searches, an additional gray literature search was performed in addition to the use of “backward snowballing” techniques. Five sources of grey literature were queried; two conventional search engines (Google, duckduckgo), two gray literature specific databases (OpenGrey and Science.gov), and the Defence Technical Information Centre (DTIC). These searches took place between January 31, 2020 and February 4, 2020. For these searches, similar terms to those used in the primary database searches were used (see supplementary file 2 for the exact syntax used for each grey literature database search). For the DTIC search, the first 100 results were investigated, while for the other grey literature sources, the first 50 were investigated (or less, if less than 50 results appeared), in a similar fashion to that discussed for Google Scholar by Bramer et al. [51]. “Backward snowballing” refers to a technique where the reference lists of previously identified reviews or journal articles within a relevant topic are searched to obtain further relevant articles [52]. Due to prior knowledge that many studies conducted in defence institutes are not published in peer-reviewed journals and are therefore not identified by primary database searches, reviews focusing on such studies were targeted for backward snowballing. Additionally, the references of two reviews on the effects of SR on cognition in the general population were also searched, as they were considered to be the closest in content to the current review. Overall, the reference sections of five reviews and one annotated bibliography were searched for relevant studies [17, 29, 53–56]. Backward snowballing was manually performed by TS. In total, 264 articles identified based on their title and abstract through the grey literature searches, and 577 articles identified through backward snowballing were screened (Figure 1).

### Eligibility criteria

The titles and abstracts obtained from the primary database search, the gray literature search, and through backward snowballing were screened and excluded only if they unambiguously did not fit the following eligibility criteria:

1. *Data.* Articles must present original data; reviews or articles representing previously available data were excluded.
2. *Population.* Participants must have been ECPs: that is, they must be members of the military (e.g. army, navy, air-force, special forces), in aviation (specifically pilot and air traffic controllers), medical personnel (physicians, surgeons, anesthesiologists, residents, etc.), alternate emergency responders (police, firefighters, etc.), process operators in a high-risk environment (i.e. mines, oil-rigs, power plants, etc.), or elite athletes (highly trained and competing regularly in their given sport). Data for participants that was influenced by the use of alcohol or psychostimulants were excluded with the exception of habitual caffeine or tobacco use.
3. *Baseline sleep condition.* Each study must have compared a SR condition to a baseline condition. This could have been conducted through a repeated measures design (each participant is exposed to both baseline and SR conditions) or an independent group design (participants exposed to a SR condition are compared to participants exposed to a baseline condition). For repeated measures designs, baseline conditions must have been conducted either before the SR condition or multiple days after SR was experienced (2-day recovery for every 1-day SR), to account for delayed recovery of cognitive performance noted following SR [24,27], where both are provided, only the baseline prior to SR condition was considered. Where a mean TST or sleep opportunity value is provided, it must be at least 6 h (TST > 6 h) and at least 2 h longer than one or more nights in the SR condition; otherwise, it must be clearly stated that baseline sleep was habitual or unhindered.
4. *Intervention.* Articles must have included SR conditions within their protocols that involved 1–7 nights whereby sleep was restricted to between 2 and 6 h of sleep opportunity or mean sleep obtained. Sleep restriction must have been either experimentally induced or resulting from an abrupt event directly causing SR to occur (e.g. 24-h overnight shift). Sleep undertaken during an overnight shift was only considered if sleep was not likely to have occurred earlier during the same day, hence 24-h shifts were considered if participants obtained some sleep throughout the night, however, night-only shifts were not. An example of a “near-miss” article that fulfilled all other criteria but was not included due to this point was by Szelenberger et al. [57]; this article was not included as it was unclear whether the sleep loss condition was due to sleep during a night-only shift allowing for prior daytime sleep or from a 24-h overnight shift. For protocols involving multiple nights of SR, all periods of sleep (sleep onset or wake time) must have commenced within the same 3-h time window within the recurring 24-h cycle. This was implemented to minimize any confounding circadian phase-shifting effects on cognitive performance [58, 59]. Similarly, any studies where protocols involved participants travelling across three or more time-zones were excluded to eliminate any confounding effects of jet-lag [60]. If multiple SR conditions were presented within the same article, only the SR condition involving night-time sleep was included. Further, daytime sleep periods were only considered if it was explicitly clear that participants were adapted to diurnal sleep prior to measurement. Sleep restriction interventions must have been monitored using sleep diaries or subjective recollections provided the day immediately following sleep, objective sleep measurement techniques (actigraphy, polysomnography (PSG), etc.), or enforced in an experimental setting through limiting sleep opportunity. If multiple sleep measurement techniques were implemented, preference for reporting sleep obtained was given to the gold standard PSG, followed by actigraphy, and finally subjective recollections.
5. *Outcome*. Articles must have evaluated cognitive performance using a validated neuropsychological test or an occupation-specific cognitively demanding task. Testing following SR interventions had to occur within the same 3-h window as testing following the baseline condition, to minimize the influence of circadian factors on performance [61]. For reviews on the effects of circadian factors on cognitive performance, see articles by Carrier and Monk [62]; Valdez et al. [63], and Van Dongen and Dinges [64]. Additionally, sufficient information must have been provided within the manuscript for each test or task (or be freely available if commonly used) to allow for classification of the test (classification described further below).

Although effect sizes are not presented within this review, they were calculated for each relevant measure. In doing so, we observed two studies presenting effect sizes on performance effects of SR that were highly improbable (hedges’ *g* >3 and greater than double the next largest effect size observed by a separate article within the test category). Due to their improbability, these two studies [65, 66] were removed from consideration in this review.

Following exclusions based on titles and abstracts, the full texts of the 802 remaining articles were screened and excluded if they did not satisfy any of the criteria described above, if articles were written in a language other than English with no translation available, or if full-texts were not present (i.e. conference abstracts). Nine corresponding authors of articles were contacted, as the results of these articles could not be included in the current state, however with clarification of population, methodologies, or results, they may have fit the criteria for the review. Unfortunately, however, only one author responded, confirming that the relevant article was not suitable for consideration here. Following full-text exclusions, 15 articles were assessed for quality.

### Quality assessment

Study quality was assessed using the specific study design tools from the National Heart, Lung, and Blood Institute (NHLBI, 2014). These criteria were chosen as the NHLBI provides multiple checklists which differ depending on study design and because they include the only standard assessment tool specifically catered for assessing repeated-measures designs within systematic reviews. These tools have been developed by expert panels, are intuitive and easy-to-use for researchers, and have been used within systematic reviews previously [67–69]. For the thirteen studies with a repeated measures design, the “Quality Assessment Tool for Before-After (Pre-Post) Studies With No Control Group” checklist was used, and for the two independent-group designs the “Quality Assessment of Controlled Intervention Studies” was used (see supplementary file 3 for the checklists in tabular form). In the latter, criteria regarding the blinding of participants to the intervention were excluded due to the practical difficulties of doing so within SR protocols. Included articles were assessed independently by TS and AT, with the agreement being reached through consensus. Using the checklists and their accompanying guidelines, articles were given a rating of “good,” “fair,” or “poor,” with “poor” articles being removed from further consideration. Overall, seven studies were assessed as “good,” eight studies were assessed as “fair,” and one study was assessed as “poor.” For the study assessed as “poor,” this was due to a >15% difference in drop-out rate between groups, constituting a “fatal flaw” and mandating a “poor” rating according to the tool [70]. This study was thus not included further in the review.

Overall, fourteen articles were included in the review (Figure 1) and were categorized according to the task used to evaluate cognitive performance (cognitive tests or performance in cognitively demanding occupation-specific tasks), as well as the risk of performance bias due to training effects.

### Test/task categorization

The tests used within each of the fourteen articles to evaluate cognitive or occupation-specific performance were categorized as “low-salience” (LS), “high-salience stable” (HSS), or “high-salience flexible” (HSF). The “low-salience” (LS) category included simple attention-based tests that involved no distractors, very limited decision-making, and typically required simple, timely responses to a stimulus. Performance was dependent on vigilance and simple attentional capacity. Low-salience tests included the psychomotor vigilance task (PVT) and serial reaction time (SRT) tests. Occupation-specific tasks were coded as “low-salience” if performance on the task primarily depended on vigilance and maintenance of simple attentional capacity. An example of such would be a vigilance rifle task, where stimuli are interspersed over very long periods of waiting, the response (shoot) is always the same, and the main determinant of performance is clearly how long the individual can maintain vigilant attention.

The “high-salience stable” (HSS) category included tests which are typically used to evaluate more complex cognitive functioning. However, performance on these tests did not depend on the ability to flexibly shift attention or adapt to changing task dynamics (i.e. cognitive flexibility). High-salience stable tests included working memory tasks, grammatical reasoning tests, and the digit symbol substitution test (DSST). Occupation-specific tasks were similarly coded as HSS if performance primarily depended on more complex cognitive functions without requiring task switching or adapting to changing dynamics. This could include psychomotor dominant tasks (skilled sports performance, surgery skill performance) as well as tasks such as friend vs foe discrimination tasks where the features discriminating friends and foes remain constant throughout.

The “high-salience flexible” (HSF) category consisted of complex/higher-salience cognitive tests & occupation-specific tasks that required cognitive flexibility and/or task switching ability for optimal performance. Examples of high-salience flexible tests included task-switching tests, multitask tests, and tests where the nature of targets could change unpredictably throughout the test. The categorization of both neuropsychological tests and ECP tasks was performed independently by two researchers (T.S. and M.K.); where there was disagreement, the consensus was reached upon consultation with AT and MC.

### Categorization of training effect bias

In order to assess the degree to which repeated-measure study designs risked confounding the effect of SR on cognitive ability by showing a training effect on cognitive performance, T.S. and N.R. reviewed the included repeated-measures design articles, rating them as having a “no risk,” “low-to-moderate risk,” or “moderate-to-high risk” of training effects, with the consensus being reached through discussion. Repeated-measures studies were considered “no risk” if the order between baseline and SR measurements was counterbalanced or if PVT was the performance outcome measure, due to thorough demonstration of the robustness of PVT to training effects [71]. Studies were considered “low-to-moderate risk” if no more than three testing sessions were administered, and “moderate-to-high” risk if more than three testing sessions were administered where the order of baseline and SR conditions were not counterbalanced between participants. Of the fifteen remaining studies, ten had “no-risk,” one had “low-to-moderate risk,” and four had “moderate-to-high” risk of training effects biasing results.

The number and age of participants in the article, occupation, nature and measurement method of SR and baseline conditions, performance test/task used, whether a significant difference was found between performance in conditions, risk of training effect bias, and quality assessment, was coded for each included article and is presented in this review. Results of the review are synthesized narratively below.

## Results

### Study characteristics

The details pertaining to each study included in this review can be found in Table 1. The 14 included articles were published between 1985 and 2019 with 13 of the articles published in peer-reviewed journals and one article published as a technical report within the Naval Medical Research Unit [72]. A total of 246 participants (41 females) were tested across all studies, with each individual study sampling 18 participants on average (*SD* = 10). The mean age of participants was not provided in each study (with some instead opting to only provide an age range), however for those that did provide this data (11 of 14), the mean age ranged from 19 to 30 years. The absence of mean age information in some studies was not considered particularly troublesome as similar effect sizes have been noted for the effects of SR on cognitive performance for individuals aged from 18 to 59 [17].

**Table 1.** Study characteristics for included articles, organized by their test/task categorization and type

Authors (year)	Population	Occupation	Test/task categorization	Cognitive test or occupation-specific task	Cognitive test used	Measure	Baseline sleep protocol	Sleep restriction (SR) protocol	Result	Risk of training effects biasing results
Englund, Ryman, Naitoh & Hodgdon (1985)	22 marine corps	Military	LS	Cognitive Test	Alpha-Numeric Visual Vigilance Task	% correct	8 h SO	3 h SO	**↓**	Moderate-to-high
					4-Choice SRT	% correct			NS	
Gillberg and Akerstedt (1994)	7 military consripts				6-minute Visual SRT	Response Time (1/RT)	8 h SO	4 h Undisturbed SO	**↓**	None
								4 h SWS-suppresed SO	**↓**	
Hartzler, Chandler, Levin & Turnmire (2015)	24 naval aviation preflight training program participants				PVT	Lapses (reaction time > 500ms)	“Unhindered sleep”	1 night 4 h SO	**↓**	None
								2 nights 4 h SO	**↓**	
								3 nights 4 h SO	**↓**	
								4 nights 4 h SO	**↓**	
						Slowest 10% Response Time (1/RT)		1 night 4 h SO	**↓**	
								2 nights 4 h SO	**↓**	
								3 nights 4 h SO	**↓**	
								4 nights 4 h SO	**↓**	
Romdhani et al. (2019)	14 elite judo athletes	Athlete			SRT	Response Time (1/RT)	8 h SO	1 night 4 h SO (early wake)	**↓**	None
								1 night 4 h SO (late sleep onset)	NS	
					2-choice SRT			1 night 4 h SO (early wake)	NS	
								1 night 4 h SO (late sleep onset)	**↓**	
Roberts, Teo, Aisbett & Warmington (2019)	9 trained cyclists or triathletes				10-minute PVT	Lapses (reaction time > 500ms)	7.1 (0.8) h sleep	1 night 4.7 h sleep	NS	None
							6.5 (1.0) h sleep	2 nights 4.7–4.8 h sleep	NS	
							6.7 (0.7) h sleep	3 nights 4.7–4.9 h sleep	**↓**	
							6.5 (1.5) h sleep	1 night 4.7 h sleep	NS	
								2 nights 4.7–4.8 h sleep	**↓**	
								3 nights 4.7–4.9 h sleep	**↓**	
						Response Time (1/RT)	7.1 (0.8) h sleep	1 night 4.7 h sleep	NS	
							6.5 (1.0) h sleep	2 nights 4.7–4.8 h sleep	NS	
							6.7 (0.7) h sleep	3 nights 4.7–4.9 h sleep	**↓**	
							6.5 (1.5) h sleep	1 night 4.7 h sleep	**↓**	
								2 nights 4.7–4.8 h sleep	**↓**	
								3 nights 4.7–4.9 h sleep	**↓**	
Mah, Sparks, Samaan, Souza & Luke (2019)	10 elite OR highly trained and actively competing cyclists				10-minute PVT	Reaction Time	7 nights of mean 6.7 (0.7) sleep	3 nights of 3.7 (0.2) h sleep	**↓**	None
						Fastest 10% Reaction Time			**↓**	
						Response Time (1/RT)			**↓**	
Saxena & George (2005)	13 medical residents	Medical			5-minute PVT	Slowest 10% Reaction Time	7.6 (3.0) h sleep	4.8 (2.4) h sleep	NS	None
						Fastest 10% Reaction Time			NS	
Sallinen et al. (2004)	12 process operators at an oil refinery	Process Operators			10-Choice SRT	Reaction Time	7.1–7.4 (0.6–0.9) h sleep	3.6–3.7 (0.1–0.2) h sleep	NS	None
						Slowest 10% Reaction Time			NS	
Haslam (1985)	6 trained infantrymen	Military		***Occupation-Specific*** Task	***Marksmanship: Vigilance*** Rifle Shooting	***Number of Hits***	2 nights 7.25 h SO	6 nights 4 h SO	NS	Moderate-to-high
Hartzler, Chandler, Levin & Turnmire (2015)	24 naval aviation preflight indoctrination program participants				***Flight Simulator***	***Total Lapse*** Time	Unhindered sleep	1 night 4 h SO	**↓**	Moderate-to-high
								2 nights 4 h SO	**↓**	
								3 nights 4 h SO	**↓**	
								4 nights 4 h SO	**↓**	
Sallinen et al. (2004)	12 process operators at an oil refinery	Process Operators			***Simulated Distillation*** Process – Monotonous Workday Simulation	***Periods of Nil*** Production	7.4 (0.6) h sleep	3.6 (0.2) h sleep	NS	None
Haslam (1985)	6 trained infantrymen	Military	HSS	Cognitive Test	Adapted Williams Word Memory Test	Number Correct	2 nights 7.25 h SO	6 nights 4 h SO	↓	Moderate-to-high
					15-minute Addition Test	Number Correct			NS	
						Number of Errors			NS	
Englund, Ryman, Naitoh & Hodgdon (1985)	22 marine corps (11 exercise, 11 non exercise)				Baddeleys Logical Reasoning Test	% correct	8 h SO	3 h SO	NS	Moderate-to-high
					Williams Auditory Word Memory Test	% correct			NS	
					Gates-Peardon Reading Exercise - “Remembering Details”	Number Correct			NS	
					Gates-Peardon Reading Exercise - “Section About”				NS	
					Gates-Peardon Reading Exercise - “Following Direction”				NS	
					Miller Reading Efficiency Test	Number of Lines Completed			NS	
Reimann, Manz, Prieur, Reichmann & Ziemssen (2009)	32 neurology residents				Paced Auditory Serial Addition Test (PASAT)	Proportion of Items Correctly Answered	6.5 (6.0–7.0) h sleep	4.3 (2.8–4.6) h sleep observed on 24 h overnight shift	NS	N/A (independent- group design)
Schlosser et al. (2012)	38 surgeons				d2-Paper-Pencil test	Main Concentration inTdex	6.7 (0.2) h sleep	4.1 (0.3) h sleep observed on 24 h overnight shift	**↑**	Low-to-moderate
Sallinen et al. (2004)	12 process operators at an oil refinery	Process Operators			Subtraction Test	Reaction Time	7.1–7.4 (0.6–0.9) h sleep	3.6–3.7 (0.1–0.2) h sleep	NS	None
						Slowest 10% Reaction Time			NS	
Haslam (1985)	6 trained infantrymen	Military		***Occupation-Specific Task***	***10-minute Map Grip Reference Encoding/ Decoding***	***Number Correct***	2 nights 7.25 h SO	6 nights 4 h SO	NS	Moderate-to-high
						***Number of Errors***			NS	Moderate-to-high
					***Marksmanship: Grouping*** Capacity	***Shooting Accuracy***			NS	Moderate-to-high
Englund, Ryman, Naitoh & Hodgdon (1985)	22 marine corps (11 exercise, 11 non exercise)				***Air Defense*** Game	***Average Range of Intercept***	8 h SO	3 h SO	NS	Moderate-to-high
Smith et al. (2019)	15 active duty soldiers				***Marksmanship: Friend vs. Foe Discrimination Task - “Low Cognitive Load (LCL)”***	***Errors (incorrect response to friend or foe target)***	7.7 (0.1) h sleep	1 Night 2 h SO	NS	Moderate-to-high
								2 Nights 2 h SO	NS	
						***Accuracy on hitting foes (%)***		1 Night 2 h SO	NS	
								2 Nights 2 h SO	NS	
					***Marksmanship: Army Record Fire Task***	***Accuracy***		1 Night 2 h SO	NS	
								2 Nights 2 h SO	NS	
Reyner and Horne (2013)	28 first or second team univeristy tennis players	Athlete			***Tennis Serving Accuracy***	***Hits Within a Designated*** Area	6.6–7.8 (SE = 0.1–0.2) h sleep	4.3–5.4 (SE = 0.1) h sleep	↓	None
Schlosser et al. (2012)	38 surgeons	Medical			***LapSim Low-Fidelity Tasks***	***Composite Performance*** Score (%)	6.7 (0.2) h sleep	4.1 (0.3) h sleep observed on 24 h overnight shift	**↑**	Low-to-moderate
					***High-Fidelity*** Intracorporeal Suturing				**↑**	
					***High Fidelity*** Chole-Cystectomy				**↑**	
Sallinen et al. (2008)	16 military conscripts	Military	HSF	Cognitive Test	Brain@work Multitask	Score obtained relative to highest possible score obtainable for the individual	8.0 (0.4) h sleep	2.1 (0.1) h sleep	**↓**	None
Hartzler, Chandler, Levin & Turnmire (2015)	24 naval aviation preflight indoctrination program participants				Dual *n*-back	Dual *n*-back Metric	Unhindered sleep	1 night 4 h SO	**↑**	Moderate-to-high
								2 nights 4 h SO	**↑**	
								3 nights 4 h SO	**↑**	
								4 nights 4 h SO	**↑**	
Smith et al. (2019)	15 active duty soldiers			***Occupation-Specific Task***	***Marksmanship: Friend vs. Foe Discrimination Task, “High Cognitive Load (HCL)”***	***Errors***	7.7 (0.1) h sleep	1 Night 2 h SO	**↓**	Moderate-to-high
								2 Nights 2 h SO	**↓**	
						***High Value*** Target Detections		1 Night 2 h SO	NS	
								2 Nights 2 h SO	**↓**	
						***Accuracy on hitting foes (%)***		1 Night 2 h SO	NS	
								2 Nights 2 h SO	NS	
Sallinen et al. (2004)	12 process operators at an oil refinery	Process Operators			***Simulated Distillation*** Process – Busy Workday Simulation	***Amount of Time with Nil*** Production	7.1 (0.9) h sleep	3.7 (0.1) h sleep	NS	None

*Note.* ↓: significant negative effect of SR condition, ↑: significant positive effect of SR condition, NS: no significant effect of SR condition. LS: low-salience, HSS: high-salience stable, HSF: high-salience flexible, PVT: psychomotor vigilance task, SRT: serial reaction test, CRT: choice reaction test, SO: sleep opportunity provided, SWS: slow-wave sleep. Variance for sleep measures is standard deviation except when specified using “SE” for standard error and is given in brackets following the value. Bolded cognitive tasks and outcomes are occupation/expertise specific performance measures

**Figure 2.**
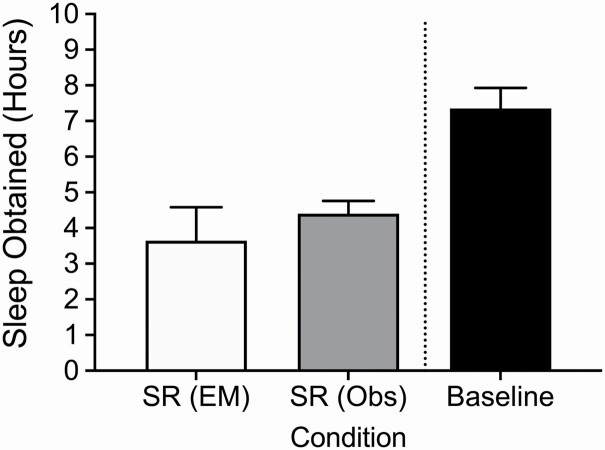
Amount of mean sleep obtained (±*SD*) within each condition. Note that for studies reporting only “sleep opportunity,” sleep obtained was considered to be the entirety of the reported period. SR = sleep restriction, EM = experimentally manipulated (*n* = 11), Obs = observed (*n* = 3).

Six studies included military personnel as participants, three tested medical service workers, four tested elite or highly-trained athletes, and one tested oil refinery process operators. Nine studies assessed performance following only 1 day of SR, one study following 2 days of SR, two studies following three days of SR, one study following 4 days of SR, and one study each following 4 days and 6 days of SR, respectively. Two studies used polysomnography (PSG) to measure the sleep of participants, five used actigraphy, two used subjective recollection, and five used enforced restriction of sleep opportunity (SO) within a laboratory. Eleven studies experimentally manipulated the SR protocol (mean reported sleep obtained across articles ≈3.6 ± 0.9 h per night SR), while the remaining three all observed sleep obtained during a 24-h overnight shift (mean reported sleep obtained across articles ≈ 4.4 ± 0.4 h per night SR). Mean baseline sleep duration was approximately 7.3 ± 0.6 h (Figure 2); note that the report by Hartzler et al. [72] was not included in this baseline mean calculation, due to reporting “unhindered” baseline sleep rather than a quantity.

Assessed using the NHLBI checklists, strengths generally included well-defined research questions, thoroughly described procedures, and minimal participant drop-out. Common weaknesses included a lack of evidence provided on the validity of performance measures and outcomes used, not providing information of whether test administrators were blinded to the condition of the participants, and a lack of consideration of statistical power when determining sample size (although in many cases, the sample size was likely limited by the availability of participants, given the specialized populations).

### Low-salience test/task performance

#### Descriptive information

Nine studies investigated “low-salience” cognitive or occupation-specific task performance following SR among 117 participants. Four of these studies tested military personnel, three tested elite or highly-trained athletes, one tested medical residents, and one tested oil refinery process operators. Five studies examined the effect of only a single night of SR on performance, while the remaining studies examined the effect of multiple consecutive nights of SR on performance. Three studies incorporated occupation-specific tasks; a marksman vigilance task, a distillation simulation task (monotonous workday condition), and a flight simulator lapse task.

#### Findings

Only performance on the flight simulator task (deviation from a “simple flight profile”) was found to be significantly weakened by SR [72]. In the other two studies, the vigilance of trained infantrymen was found to be unaffected while performing a shooting task following six consecutive nights of SR (4 h SO) [73], and no significant performance change was found on a simulated distillation task among experienced oil-refinery process operators following one night of ~3.5 h TST [74]. Among the eight studies testing cognitive abilities directly, Englund et al. [75] observed a significant performance decrement among a sample of U.S. marines on the alpha-numeric visual vigilance task, but not on a four-choice SRT, following one night of SR (3 h SO). Gillberg and Åkerstedt [76] found response times on a SRT were not significantly affected by SR when tested at 08:00 am, but were significantly worsened when tested at 14:00 pm or 20:30 pm, as well as when the results from all time points were combined; this was regardless of whether the 4 h of SO allocated were undisturbed or manipulated so that participants could obtain minimal slow-wave sleep. Among a sample of naval aviation trainees, Hartzler et al. [72] found the number of lapses (reaction time > 500 ms) increased during each night of SR experienced (four nights of 4 h SO), with an increased overall response time of the slowest 10% attempts on the PVT compared to baseline following SR. Romdhani et al. [77] found that one night of SR (4 h SO) slowed reaction times of judo athletes on (1) a SRT when sleep was restricted by initiating an early wake time but not when delaying sleep onset, and (2) a “choice” reaction time task when sleep was restricted by delaying sleep onset but not when initiating an early wake time. Roberts et al. [78] found a significant increase in PVT lapses and response times among highly trained cyclists and triathletes following three days of SR (~4.5–5 h TST) when compared both to the day before the first night of SR (6.5 h TST) or the equivalent day within a baseline condition (~6.5–7 h TST), additionally finding differences in lapses following 2 days of SR and in response times following both 1 and 1 days of SR when compared to the equivalent baseline condition. Mah et al. [79] similarly found the vigilance of 10 elite (or highly trained & actively competing) cyclists (PVT reaction time, inverse reaction time & fastest 10% reaction time) to be adversely affected by three nights of ~4 h SO. Interestingly, no significant differences in LS test performance (PVT, serial reaction time task) were found between SR (~5 h TST) and baseline conditions for medical residents [80], nor among oil refinery process operators following one night of ~3.5 h TST [74].

### High-salience stable test/task performance

#### Descriptive information

Seven studies examined the effects of SR on cognitive and occupation-specific “high-salience stable” tasks among 153 participants (Table 1). Three of these studies tested military personnel, one tested highly-trained athletes, two tested surgeons or medical residents, and one tested oil refinery process operators. Five studies implemented only a single night of SR, while two involved multiple consecutive nights of SR. Five studies incorporated occupation-specific tasks, including marksmanship accuracy tasks, a marksmanship friend vs foe discrimination task (low-cognitive load condition), an “air defense” game, a map-grip encoding/decoding task, a tennis serving accuracy protocol, and a VR-surgery simulator task.

#### Findings

Two studies found SR to significantly decrease performance relative to a baseline condition. Haslam [73] found significant deterioration in the number of correctly recalled items by trained infantrymen on a word memory test throughout six nights of SR (4 h SO). Conversely, Englund et al. [75] found no effect of one night of SR (3 h SO) on the immediate recall of marine corps on an almost identical task to that in Haslam [73], as well as the ability to immediately recall details in a short reading task. Performance on the d2-paper-pencil test (selective attention) was found to significantly improve following SR (one night of ~4 h TST) relative to a previously taken baseline among surgeons [47]. This study additionally found performance on surgery skills of varying complexity (as well as “economy of motion” measures which comprised these overall composite scores) on a VR-surgery simulator to *improve* following SR. It is noted however that in measurements taken 24 h after the SR condition testing, d2-paper-pencil test performance improved again from the SR condition, and performance on two of the three surgery performance measures (Low-Fidelity Task and Chole-Cystectomy performance score) was maintained from the SR condition and also significantly better than in the baseline condition. When examining studies that evaluated occupation-specific task performance, Reyner and Horne [81] found serving accuracy of semi-elite tennis players to be hindered by one night of SR (~4.5–5.5 h TST), whereas all other studies utilizing occupation-specific HSS measures failed to detect significant performance differences between baseline and SR conditions.

### High-salience flexible test/task performance

#### Descriptive information

Four studies examined performance outcomes on “high-salience flexible” cognitive and occupation-specific tasks among 67 participants. Three of these studies tested military personnel, and one tested oil refinery process operators. Two of these studies investigated only a single night of SR, while the other two implemented multiple consecutive nights of SR. Two studies incorporated occupation-specific tasks including a military marksmanship friend vs foe discrimination task (high-cognitive load condition), and an oil-refinery distillation simulation task, similar to the task mentioned previously but with increased cognitive demand and functional instability embedded within it.

#### Findings

Among those studies investigating cognitive test performance, Sallinen et al. [82] tested 16 military conscripts on a multitask test (Brain@Work) involving four cognitive tests performed simultaneously, and reported a significant decrease in performance following SR (one night ~2 h TST) relative to baseline. Alternatively, Hartzler et al. [72] used an adaptive dual *n*-back measure requiring simultaneous attention toward both visual and auditory stimuli, and found *improvement* compared to baseline on all 4 days of SR (4 h SO). However, the authors concluded that “practice effects were evident throughout the study” (p. 24) and these likely confounded the results. For studies investigating occupation-specific performance, Smith et al. [83] found one and two nights of SR (2 h SO) led to an increase in the number of errors made on a high cognitive load (HCL) challenge of the marksmanship friend vs foe discrimination task. In this task, colors that represented friends and foes changed frequently, requiring participants to flexibly adapt to the task details for correct completion. Notably, in the low cognitive load (LCL) version of this challenge, whereby color coding was held constant (HSS task), error rate did not significantly differ between baseline and SR conditions. Additionally, for high-value target detection, a measure presents only for the HCL version of the task, the percentage detection rate was impaired after two nights of SR. No effect of SR was found in either version of the task for the marksmanship accuracy in shooting foes. Lastly, time at nil production, the main performance outcome referring to a lack of activity occurring within the “busy” condition of a simulated distillation process, was not found to change significantly between SR (one night of ~3.5 h TST) and baseline conditions for oil refinery process operators [74]. Again, this task was identical to the “monotonous” condition in the simulated distillation included in the LS table except for a greatly increased depletion speed and the addition of functional instability.

## Discussion

Our systematic review explored the literature examining the effect of SR on cognitive and task-related performance specifically among *Elite Cognitive Performers* (individuals within occupations that (1) have cognitive demands exceeding the norm and (2) have critical outcomes associated with these demands). In doing so, we aimed to provide an indication of how SR may affect the cognitive performance of those for whom the outcomes are arguably more important than is often the case within the general population, with a degree of specificity not previously available within the literature. Overall, this review found that the performance of this select group on monotonous, low-salience tasks is often poorer following SR, and while performance on more complex, yet cognitively stable, tasks are usually maintained, performance may be more prone to decline when the task involves adaptation to changing goal-oriented information and a shifting of attention.

### Differences of the effects of sleep restriction as a function of task demands

In this review, we found performance on simple tests designed to measure vigilance and rudimentary attentional capacity (whether occupation-specific or not) was most commonly hindered by SR. This corroborates findings from a meta-analysis on the wider population [17] and is consistent with the effects found following TSD [16]. The ability to maintain attention in low-salience circumstances is integral to many components of safety-critical work (i.e. monitoring human–machine interfaces or environment), however, two of the three studies using low-salience occupation-specific task performance found no significant deterioration resulting from SR. These two articles however had factors within their design or within the outcomes themselves that could have confounded results. Specifically, Sallinen et al. [74], who tested performance on a simulated distillation task among oil refinery workers, reported that their failure to find an effect of one night of SR on participants’ “monotonous” and “busy” workday (the latter coded as HSF) may be due to the performance task being “too rough to demonstrate a significant sleep debt-related effect” (p. 293). The other task was a vigilance rifle shooting task for trained infantrymen following six nights of SR [73]. This article stated that the infantrymen were to receive long-weekend leave if they “maintained a certain standard” of performance on “key tasks” (p. 91), likely raising the external motivation to maintain performance in spite of SR (although it is unclear which were the “key tasks” in this study). Such external motivation likely increased task engagement, which purportedly facilitates the maintenance of performance in spite of sleep loss [37]. Along these same lines, one of the few studies that did not find a difference in PVT performance following SR rewarded the highest-performing participants with $100CAD [80]. Hence, it appears that context and motivation are key moderators of the effect of SR on the performance of simpler tasks; if task engagement is promoted (through task demand, external motivation, etc.) performance is likely to be maintained, whereas if there is a lack of exogenous factors promoting task engagement, ECPs, like the general population, will show degraded performance on tasks prioritizing simple attention.

In stark comparison to studies investigating the effect of SR on simple cognitive performance, studies using evaluation tools that required more complex cognitive processes that rewarded cognitive stability *almost unanimously* reported no effect of SR. The two studies which reported SR to negatively affect performance tested the immediate recall of infantrymen on a working memory task [73], and university representative tennis players on a tennis serving accuracy test respectively [81]. The former suggests that immediate recall is vulnerable to SR, however, it is to be noted that the immediate recall of marine corps in a separate study [75] with a similar procedure to Haslam [73], as well as the ability to immediately recall details in a short reading task, was not significantly affected by SR. The latter finding would suggest perhaps that skilled, psychomotor performance outcomes could be vulnerable to SR, however, Schlosser and colleagues [47] contrastingly noted an *increase* in performance on simulated surgery tasks of differing complexity following SR and compared to a previously taken baseline condition. Investigating tasks with similar cognitive demands, Haslam [73] and Smith et al. [83] did not report a significant effect of six or two nights of SR respectively on marksmanship tasks specifically measuring accuracy. Taken altogether, these results suggest that acute SR alone is unlikely to negatively affect the performance of ECPs on complex cognitively stable tasks when they are related to their area of expertise. In a practical sense, this could be interpreted to closed skills for elite athletes (i.e. a free-throw or a golf putt), the performance of routine fine-motor, yet demanding surgery task, or the ability to perform fixed and predictable tasks such as adhering to correct pre-flight procedures for an aircraft pilot. Lastly, in further support of the influence of context, engagement, and motivation, Englund et al. [75] noted in discussing the (statistically insignificant) trend of performance improvement on a complex “air defense” game and the lack of performance loss on a reading efficiency following SR, that “competition and interest, each a motivating factor, influenced both psychomotor and cognitive task performance” (p. 84).

We separated “HSS” and “HSF” based on whether the task demanded *cognitive flexibility*; that is, the ability to shift attention to new, more relevant information, or to adapt to a changing task dynamic [84, 85]. Here, we found that performance on a multitask test [82] and error rate within a task embedded within a marksmanship context [83], both of which required cognitive flexibility, were negatively affected by SR. The latter is particularly notable as error rate in a simpler adaptation of the same task, which didn’t require participants to adapt to the changing meaning of different color targets throughout, was not negatively affected by the same conditions of SR. When considering the two studies that did *not* find a negative effect of SR on HSF outcomes, one used an adapted version of the aforementioned oil refinery distillation task with potential sensitivity issues, and the other one had a “moderate-to-high” risk of bias, with the authors [72] themselves stating that “practice effects were evident” (p. 24). The ability to flexibly shift attention and adapt to changing dynamics is of obvious ecological importance, particularly for safety-critical workers handling emergency situations. For example, aircraft pilots are presented with a multitude of information from dials, outputs, and air-traffic controllers when in an emergency situation (i.e. engine failure) and must be able to rapidly shift their focus to the most important interface to gather the most relevant information for the resolution of their current circumstance. Pilots must then be able to adapt to their new situation (flying an aircraft without the engine) and adjust their approach accordingly. Further experimental work is clearly required to understand how the cognitive flexibility of ECPs’ is affected by SR and how this impacts high-demand tasks within their workplace, given (1) the importance of cognitive flexibility particularly within emergency scenarios, (2) the increased prevalence of SR in ECPs versus the general population, and (3) the studies detailed within this review outlining the effects of SR on the cognitive flexibility of ECPs.

### Strengths and limitations

**Figure 3.**
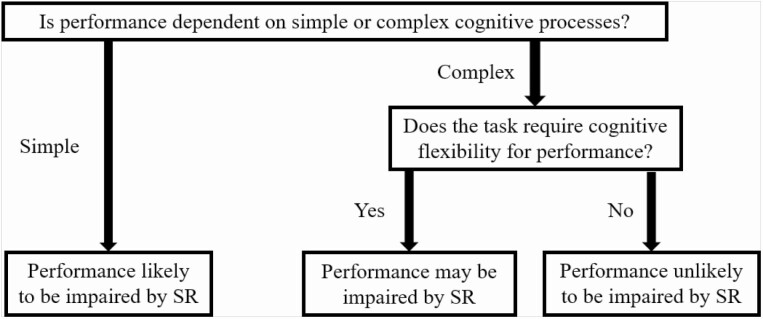
Proposed framework explaining the likelihood of sleep restriction affecting cognitive performance for Elite Cognitive Performers.

It is accepted that the classification of performance tests in this review can be considered coarse. For example, standardized cognitive tests within the HSS category can be attempting to test primarily inhibition, working memory, decision-making, executive functioning, “complex attention,” “cognitive throughput,” and so on. These are regularly discussed as distinct cognitive outcomes with distinguishable underlying neural processes. Although previous meta-analyses have demonstrated differences in effect sizes among different “complex” cognitive domains [16, 17], the most tangible distinctions regarding effects of sleep loss on cognitive performance appear to be: (1) the extent to which performance is dependent on sustained, simple attention [15–17, 37], and more recently, and (2) whether cognitive flexibility is prioritized over cognitive stability for performance [38, 86, 87] (spawning the rationale behind the classification used). This information is of direct practical use to members of safety-critical industries, elite athletes and coaches, and other individuals in occupations with cognitive demands spanning beyond the norm. The separation of tasks into cognitive domains limits applicability because tasks that ECPs engage in are complex by nature and require significant contributions from multiple domains simultaneously. By separating tasks as we have in the current review, we provide a simple framework that applies to “real-world” tasks in a host of occupations with large cognitive demands, but that seemingly distinguishes between tasks in which performance is likely or unlikely to be affected by SR (Figure 3).

The current review had particularly stringent eligibility criteria. Studies that investigated sleep restriction and cognitive performance among ECPs, but that allowed significant variance (or lacked reporting) of participant sleep onset & wake times, or testing times, were removed. Additionally, multiple studies included in this review presented additional measurements taken and statistical comparisons made that were not included due to these reasons [72, 73]. By removing these studies and measurements we likely lost some degree of ecological validity as SR in practice is often accompanied by a shift in sleep onset and wake times (i.e. following transmeridian travel, change from day-shifts to night-shifts etc.) resulting in circadian desynchrony, and performance at different and often rapidly changing times of the day is necessary (i.e. rotating shift schedules). The effect of rhythmic fluctuations of performance related to circadian rhythm, as well as the desynchronization of circadian rhythm likely to arise from large variance in sleep onset and wake time, is nontrivial [61, 62, 64, 88]. Therefore, without controlling for changes based on when the participants were sleeping or when they were being tested, it would be incredibly difficult to discern whether differences in performance were due to the changes in sleep quantity or these circadian factors. Controlling for these factors allows us to more confidently conclude that any performance decrements observed were due to SR and not other influences. In short, this review only examined the effects directly related to change in sleep quantity, and that other features commonly experienced with SR such as shifts in sleep periods are likely to further exacerbate the performance impairments discussed in this review; hence, the findings of this review should be considered conservative and a “best case outcome” for how moderate sleep loss impacts task performance for Elite Cognitive Performers in the real-world.

Despite the stringency of the eligibility criteria, there was still a surprisingly small number of articles that met the inclusion criteria of the review, given the exhaustive nature of our systematic search. In particular, there was a dearth of research investigating the role of SR on cognitive performance among elite athletes. Using the criteria for defining and quantifying expertise as outlined by Swann et al. [89], “semi-elite” athletes were the top-level participants tested among the included studies. Some of the other studies utilized interns and junior medical residents [47, 80] and military personnel either within their first few years of service or within the process of completing specialized programs such as a naval preflight training program [72,83], and may not be representative of more experienced individuals who (1) have more experience performing while fatigued, and (2) have greater expertise on occupation-specific tasks. This distinction is highly important as more expert individuals tend to utilize different cognitive strategies compared to their less-skilled individuals when performing tasks within their field of expertise, such as different gaze and fixation strategies [90] and decision-making processes [91]. Hence, it is possible that experts and novices may be differentially affected by sleep loss on a particular cognitively demanding task. Ideally, further experimental work which directly investigates the potential for expertise to moderate the effect of SR on cognitive performance would elucidate such a possibility. However, recruiting such an array of participants within a specific area can prove difficult. Additionally, as demonstrated by Sallinen et al. [74], selecting a performance outcome within the context of one's area of cognitive expertise that is also sensitive enough to show performance deficits following SR provides another layer of difficulty. Still, such experimental work could be extremely beneficial in (1) understanding the relationships between sleep loss, cognitive performance, and cognitive expertise, and (2) further improving our overall understanding on how SR affects the task performance of ECPs.

### Future directions

One area where it is both relatively easy to evaluate cognitive and occupational performance among individuals with a vast array of skill level is esports. Here, we believe that research on elite esports athletes may be able to provide insight into the moderating effect of cognitive expertise on performance loss resulting from SR. Esports refers to the competitive (and for some, professional) play of commercially available video games, with esports athletes being referred to as “cognitive athletes” due to the cognitive expertise that they possess [92]. Many esports games often adopt the Elo rating system, allowing for expertise to be quantified on a continuum and the digital nature of gameplay facilitates the collection of large amounts of relevant performance. In addition to being an exemplar test population, esports athletes also share many similarities with many ECPs with respect to the work environment and the enhanced cognitive skills required by both for optimal performance [93]. Future research on the effects of SR on esport athletes could thus provide applicability to ECPs in general, furthering our understanding of how elite cognitive performers are affected by sleep loss. Moreover, as esport athletes can be considered ECPs themselves and that their shared commonalities with traditional athletes likely leading to the higher-than-normal prevalence of SR, the results of the current review are of great relevance to this population.

Sleep restriction presents as one of many factors which may adversely affect performance on complex, cognitively demanding tasks. In addition to circadian factors mentioned earlier, sleep inertia, referring to the grogginess and degraded performance immediately following wake, is of high relevance to individuals performing tasks at night or those working extended shifts and are able to sleep on the job but simultaneously may be required to respond to complex emergency situations at a moment’s notice (i.e. night-shift medical workers, pilots, air traffic controllers, emergency responders). Extended periods of wakefulness and time on task (particularly for boring, monotonous tasks) can also further contribute to fatigue-related performance impairment within the workplace [94], and are important considerations for safety-critical workers and other elite cognitive performers (i.e. athletes, esport athletes). As aforementioned, the context surrounding a given task (i.e. the presence of external motivating factors) is an important consideration in addition to the nature and demands of the task itself. Lastly, the extension of sleep quantity beyond what is habitually obtained has shown positive effects on cognitive performance outcomes for high-level collegiate athletes measured both through standardized cognitive tests and through outcomes directly related to their expertise [95], and may resemble a fruitful strategy to improve performance on demanding tasks for Elite Cognitive Performers overall.

## Conclusion

In summary, the current review demonstrates that the performance of ECPs is more negatively affected on simple cognitive tests and monotonous occupation-specific tasks, where simple attentional capabilities are instrumental to task success, over more complex cognitive tasks; however, performance may be more affected on complex tasks when adaptation to changing goal-oriented information and a shifting of attention (i.e. cognitive flexibility). Further research is required particularly when using tasks demanding cognitive flexibility as there is little and conflicting evidence on the effect of SR on the performance of such tasks. Lastly, we believe that esports presents as a fruitful medium to explore the effects of sleep loss on *Elite Cognitive Performers*, potentially uncovering moderating roles of expertise and providing applicability to many industries and occupations.

## Supplementary Material

zsab008_suppl_Supplementary_Material
