# Supplementary material for: The effect of sleep restriction on cognitive performance in elite cognitive performers: a systematic review
Source: Sleep. 2021 Jan 13;44(7):zsab008. doi: 10.1093/sleep/zsab008 (PMC8271199; doi:10.1093/sleep/zsab008)
Supplement: zsab008_suppl_Supplementary_Material [file zsab008_suppl_supplementary_material.docx]

**The Effect of Sleep Restriction on Cognitive Performance in Elite Cognitive Performers: A Systematic Review**

Tim D. Smithies^1, 2^, Adam J. Toth^1, 2^, Ian C. Dunican^3, 4^, John A. Caldwell^5^, Magdalena Kowal^1, 2^ & Mark J. Campbell^1, 2 *^

TS - [0000-0002-8026-5134](http://orcid.org/0000-0002-8026-5134)

AT – 0000-0003-2193-0138

ID – 0000-0002-4000-8213

JC – 0000-0001-5281-0820

MK – 0000-0002-4768-4900

MC – 0000-0001-9607-7675

*^1^ Department of Physical Education & Sport Science, University of Limerick, Castletroy, Limerick, Ireland*

*^2^ Lero, the SFI Centre for Software Research, University of Limerick, Castletroy, Limerick, Ireland*

*^3^ Melius Consulting, Mount Hawthorn, Western Australia, Australia*

*^4^ Centre for Sleep Science, School of Human Sciences, University of Western Australia, Crawley, Western Australia, Australia*

*^5^ Coastal Performance Consulting, United States*

* Corresponding Author: Tim D. Smithies^1,2^

Department of Physical Education and Sport Sciences,

Lero, the SFI Centre for Software Research,

University of Limerick, Ireland

[Tim.Smithies@ul.ie](mailto:Tim.Smithies@ul.ie) | +353899424679

Supplementary File 1: The exact syntax used for each primary database

EMBASE

(((motor OR skill OR performance OR psychomotor OR sensorimotor OR visuomotor OR neuropsychological OR visuospatial OR visual OR cogniti* OR executive function* OR problem solving OR spatial OR verbal OR learning OR percept* OR attention OR ‘reaction time’ OR ‘response time’ OR vigilan* OR neurocognitive):ab,ti) OR (‘psychomotor performance’/exp OR ‘neuropsychological test’/exp OR ‘cognition’/exp OR ‘reaction time’/exp OR ‘motor performance’/exp OR ‘motor control’/exp OR ‘motor learning’/exp OR ‘sensorimotor function’/exp)) AND ((‘sleep restriction’ OR ‘partial sleep deprivation’ OR ‘sleep manipulation’ OR ‘sleep loss’ OR ‘sleep debt’):ab,ti) OR (‘sleep debt’/exp) NOT ((‘mice’ OR ‘mouse’ OR ‘rat*’ OR ‘rodent*’ OR ‘monkey*’ OR ‘cat’ OR ‘dog’ OR ‘drosophila’ OR ‘cognitive behavioural therapy’ OR ‘CBT’ OR ‘depression’ OR ‘syndrome’ OR ‘disorder*’ OR ‘cognitive impairment’ OR ‘cognitive decline’ OR ‘insomnia*’ OR ‘narcolep*’ OR ‘bruxism’ OR ‘alzheimer*’ OR ‘schizophrenia’ OR ‘epilep*’ OR ‘apnea’ OR ‘apnoea’ OR ‘obesity’ OR ‘diabet*’ OR ‘pregnan*’ OR ‘review’):ti)

OVID MEDLINE(R) AND EPUB AHEAD OF PRINT, IN-PROCESS & OTHER NON-INDEXED CITATIONS, DAILY AND VERSIONS(R)

(((motor or skill or performance or psychomotor or sensorimotor or visuomotor or neuropsychological or visuospatial or visual or cogniti* or executive function* or problem solving or spatial or verbal or learning or percept* or attention or reaction time or response time or vigilan* or neurocognitive).ab,ti.) OR (exp psychomotor performance/ or exp neuropsychological tests/ or exp mental processes/ or exp reaction time/ or exp motor activity/)) AND (sleep restriction or partial sleep deprivation or sleep manipulation or sleep loss or sleep debt).ab,ti. NOT (sleep restriction or partial sleep deprivation or sleep manipulation or sleep loss or sleep debt).ab,ti. not (mice or mouse or rat* or rodent* or monkey* or cat or dog or drosophila or cognitive behavioural therapy or CBT or depression or syndrome or disorder* or cognitive impairment or cognitive decline or insomnia* or narcolep* or bruxism or alzheimer* or schizophrenia or epilep* or apnea or apnoea or obesity or diabet* or pregnan* or review).ti.

Web of Science (Core Collection)

((TS=(motor OR skill OR performance OR psychomotor OR sensorimotor OR visuomotor OR neuropsychological OR visuospatial OR visual OR cogniti* OR executive function* OR problem solving OR spatial OR verbal OR learning OR percept* OR attention OR “reaction time” OR “response time” OR vigilan* OR neurocognitive)) AND (TS=(“sleep restriction” OR “partial sleep deprivation” OR “sleep manipulation” OR “sleep loss” OR “sleep debt”))) NOT (TI=(mice OR mouse OR rat* OR rodent* OR monkey* OR cat OR dog OR drosophila OR “cognitive behavioural therapy” OR CBT OR depression OR syndrome OR disorder* OR “cognitive impairment” OR “cognitive decline” OR insomnia* OR narcolep* OR bruxism OR alzheimer* OR schizophrenia OR epilep* OR apnea OR apnoea OR obesity OR diabet* OR pregnan* OR review))

Google Scholar

(performance | motor | skill | psychomotor | neuropsychological | visual | cognitive | executive function | spatial | verbal | learning | perception | attention | reaction time | vigilance) (sleep restriction | partial sleep deprivation | sleep loss)

APA PSYCinfo (EBSCO host)

(((TI (motor) OR AB (motor) OR TI (skill) OR AB (skill) OR TI (performance) OR AB (performance) OR TI (psychomotor) OR AB (psychomotor) OR TI (sensorimotor) OR AB (sensorimotor) OR TI (visuomotor) OR AB (visuomotor) OR TI (neuropsychological) OR AB (neuropsychological) OR TI (visuospatial) OR AB (visuospatial) OR TI (visual) OR AB (visual) OR TI (cogniti*) OR AB (cogniti*) OR TI (executive function*) OR AB (executive function*) OR TI (problem solving) OR AB (problem solving) OR TI (spatial) OR AB (spatial) OR TI (verbal) OR AB (verbal) OR TI (learning) OR AB (learning) OR TI (percept*) OR AB (percept*) OR TI (attention) OR AB (attention) OR TI (“reaction time”) OR AB (“reaction time”) OR TI (“response time”) OR AB (“response time”) OR TI (vigilan*) OR AB (vigilan*) OR TI (neurocognitive) OR AB (neurocognitive)) OR (MA “psychomotor performance” OR MA “neuropsychological tests” OR MA “mental processes” OR MA “reaction time” OR MA “motor activity”)) AND ((TI (“sleep restriction”) OR AB (“sleep restriction”) OR TI (“partial sleep deprivation”) OR AB (“partial sleep deprivation”) OR TI (“sleep manipulation”) OR AB (“sleep manipulation”) OR TI (“sleep loss”) OR AB (“sleep loss”) OR TI (“sleep debt”) OR AB (“sleep debt”)) NOT (TI (mice) OR TI (mouse) OR TI (rat*) OR TI (rodent*) OR TI (monkey*) OR TI (cat) OR TI (dog) OR TI (drosophila) OR TI (“cognitive behavioural therapy”) OR TI (CBT) OR TI (depression) OR TI (syndrome) OR TI (disorder*) OR TI (“cognitive impairment”) OR TI (“cognitive decline”) OR TI (insomnia*) OR TI (narcolept*) OR TI (bruxism) OR TI (alzheimer*) OR TI (schizophrenia) OR TI (epilep*) OR TI (apnea) OR TI (apnoea) OR TI (obesity) OR TI (diabet*) OR TI (pregnan*) OR TI (review))

SPORTDiscus with full text (EBSCO host)

(((TI (motor) OR AB (motor) OR TI (skill) OR AB (skill) OR TI (performance) OR AB (performance) OR TI (psychomotor) OR AB (psychomotor) OR TI (sensorimotor) OR AB (sensorimotor) OR TI (visuomotor) OR AB (visuomotor) OR TI (neuropsychological) OR AB (neuropsychological) OR TI (visuospatial) OR AB (visuospatial) OR TI (visual) OR AB (visual) OR TI (cogniti*) OR AB (cogniti*) OR TI (executive function*) OR AB (executive function*) OR TI (problem solving) OR AB (problem solving) OR TI (spatial) OR AB (spatial) OR TI (verbal) OR AB (verbal) OR TI (learning) OR AB (learning) OR TI (percept*) OR AB (percept*) OR TI (attention) OR AB (attention) OR TI (“reaction time”) OR AB (“reaction time”) OR TI (“response time”) OR AB (“response time”) OR TI (vigilan*) OR AB (vigilan*) OR TI (neurocognitive) OR AB (neurocognitive)) OR (MA “psychomotor performance” OR MA “neuropsychological tests” OR MA “mental processes” OR MA “reaction time” OR MA “motor activity”)) AND ((TI (“sleep restriction”) OR AB (“sleep restriction”) OR TI (“partial sleep deprivation”) OR AB (“partial sleep deprivation”) OR TI (“sleep manipulation”) OR AB (“sleep manipulation”) OR TI (“sleep loss”) OR AB (“sleep loss”) OR TI (“sleep debt”) OR AB (“sleep debt”)) NOT (TI (mice) OR TI (mouse) OR TI (rat*) OR TI (rodent*) OR TI (monkey*) OR TI (cat) OR TI (dog) OR TI (drosophila) OR TI (“cognitive behavioural therapy”) OR TI (CBT) OR TI (depression) OR TI (syndrome) OR TI (disorder*) OR TI (“cognitive impairment”) OR TI (“cognitive decline”) OR TI (insomnia*) OR TI (narcolept*) OR TI (bruxism) OR TI (alzheimer*) OR TI (schizophrenia) OR TI (epilep*) OR TI (apnea) OR TI (apnoea) OR TI (obesity) OR TI (diabet*) OR TI (pregnan*) OR TI (review))

Supplementary File 2: the exact syntax used for each grey literature database search

Google Search

(performance | motor | skill | psychomotor | neuropsychological | visual | cognitive | executive function | spatial | verbal | learning | perception | attention | reaction time | vigilance) (sleep restriction | partial sleep deprivation | sleep loss)

Duckduckgo Search

(performance | motor | skill | psychomotor | neuropsychological | visual | cognitive | executive function | spatial | verbal | learning | perception | attention | reaction time | vigilance) (sleep restriction | partial sleep deprivation | sleep loss)

OPENGREY

(motor OR skill OR performance OR psychomotor OR sensorimotor OR visuomotor OR neuropsychological OR visuospatial OR visual OR cogniti* OR executive function* OR problem solving OR spatial OR verbal OR learning OR percept* OR attention OR ‘reaction time’ OR ‘response time’ OR vigilan* OR neurocognitive) AND (‘sleep restriction’ OR ‘partial sleep deprivation’ OR ‘sleep manipulation’ OR ‘sleep loss’ OR ‘sleep debt’)

Defense Technical Information Center (DTIC)

(performance OR motor OR psychomotor OR neuropsychological OR visual OR cognitive OR executive function OR spatial OR verbal OR learning OR perception OR attention OR reaction time OR vigilance) AND (sleep restriction OR partial sleep deprivation)

Science.gov

(performance OR motor OR psychomotor OR neuropsychological OR visual OR cognitive OR executive function OR spatial OR verbal OR learning OR perception OR attention OR reaction time OR vigilance) AND (sleep restriction OR partial sleep deprivation)

Supplementary File 3: NHLBI checklists in tabular form

| **Quality Assessment Tool for Before-After (Pre-Post) Studies with No Control Groups** |  |  |  |
| --- | --- | --- | --- |
| **Criteria** | **Yes** | **No** | **Notes** |
| Was the study question or objective clearly stated? |  |  |  |
| Were eligibility/selection criteria for the study population prespecified and clearly described? |  |  |  |
| Were the participants in the study representative of those who would be eligible for the test/service/intervention in the general or clinical population of interest? |  |  |  |
| Were all eligible participants that met the prespecified entry criteria enrolled? |  |  |  |
| Was the sample size sufficiently large to provide confidence in the findings? |  |  |  |
| Was the test/service/intervention clearly described and delivered consistently across the study population? |  |  |  |
| Were the outcome measures prespecified, clearly defined, valid, reliable, and assessed consistently across all study participants? |  |  |  |
| Were the people assessing the outcomes blinded to the participants' exposures/interventions? |  |  |  |
| Was the loss to follow-up after baseline 20% or less? Were those lost to follow-up accounted for in the analysis? |  |  |  |
| Did the statistical methods examine changes in outcome measures from before to after the intervention? Were statistical tests done that provided p values for the pre-to-post changes? |  |  |  |
| Were outcome measures of interest taken multiple times before the intervention and multiple times after the intervention (i.e., did they use an interrupted time-series design)? |  |  |  |
| If the intervention was conducted at a group level (e.g., a whole hospital, a community, etc.) did the statistical analysis take into account the use of individual-level data to determine effects at the group level? |  |  |  |
| **Quality Assessment Tool for Controlled Intervention Studies** |  |  |  |
| **Criteria** | **Yes** | **No** | **Notes** |
| Was the study described as randomized, a randomized trial, a randomized clinical trial, or an RCT? |  |  |  |
| Was the method of randomization adequate (i.e., use of randomly generated assignment)? |  |  |  |
| Was the treatment allocation concealed (so that assignments could not be predicted)? |  |  |  |
| ~~Were study participants and providers blinded to treatment group assignment?~~ |  |  |  |
| Were the people assessing the outcomes blinded to the participants' group assignments? |  |  |  |
| Were the groups similar at baseline on important characteristics that could affect outcomes (e.g., demographics, risk factors, co-morbid conditions)? |  |  |  |
| Was the overall drop-out rate from the study at endpoint 20% or lower of the number allocated to treatment? |  |  |  |
| Was the differential drop-out rate (between treatment groups) at endpoint 15 percentage points or lower? |  |  |  |
| Was there high adherence to the intervention protocols for each treatment group? |  |  |  |
| Were other interventions avoided or similar in the groups (e.g., similar background treatments)? |  |  |  |
| Were outcomes assessed using valid and reliable measures, implemented consistently across all study participants? |  |  |  |
| Did the authors report that the sample size was sufficiently large to be able to detect a difference in the main outcome between groups with at least 80% power? |  |  |  |
| Were outcomes reported or subgroups analyzed prespecified (i.e., identified before analyses were conducted)? |  |  |  |
| Were all randomized participants analyzed in the group to which they were originally assigned, i.e., did they use an intention-to-treat analysis? |  |  |  |
| Note: details of each question, as well as guidelines for determining overall study quality, can be found at https://www.nhlbi.nih.gov/health-topics/study-quality-assessment-tools |  |  |  |
